# Weight and Height in Children and Adolescents with Attention-Deficit/Hyperactivity Disorder: A Longitudinal Database Study Assessing the Impact of Guanfacine, Stimulants, and No Pharmacotherapy

**DOI:** 10.1089/cap.2018.0132

**Published:** 2019-05-13

**Authors:** Gary Schneider, Tobias Banaschewski, Brian L. Feldman, Per A. Gustafsson, Brian Murphy, Matthew Reynolds, David R. Coghill, William M. Spalding

**Affiliations:** ^1^ Evidera, Waltham, Massachusetts.; ^2^ Department of Child and Adolescent Psychiatry and Psychotherapy, Central Institute of Mental Health, Medical Faculty Mannheim, University of Heidelberg, Mannheim, Germany.; ^3^ Department of Pediatrics, Naval Medical Center Portsmouth, Portsmouth, Virginia.; ^4^ Center for Social and Affective Neuroscience, Department of Clinical and Experimental Medicine and Department of Child and Adolescent Psychiatry, Linköping University, Linköping, Sweden.; ^5^ Departments of Pediatrics and Psychiatry, Faculty of Medicine, Dentistry and Health Sciences, University of Melbourne, Melbourne, Australia.; ^6^ Murdoch Children's Research Institute, Melbourne, Australia.; ^7^ Division of Neuroscience, University of Dundee, Dundee, United Kingdom.; ^8^ Shire, now part of Takeda, Lexington, Massachusetts.

**Keywords:** attention-deficit/hyperactivity disorder, weight, height, guanfacine, stimulant

## Abstract

***Objectives:*** To assess the impact of long-term pharmacotherapy with guanfacine immediate- or extended-release (GXR), administered alone or as an adjunctive to a stimulant, on weight and height in children and adolescents with attention-deficit/hyperactivity disorder (ADHD).

***Methods:*** Data were extracted from U.S. Department of Defense medical records for patients 4–17 years of age at index date (initiation of any study medication following a year without ADHD medications, or diagnosis if unmedicated) with weight/height measurements for the analysis period (January 2009–June 2013) and the previous year (baseline). Longitudinal weight and height *z*-scores were analyzed using multivariable regression in three cohorts: guanfacine (initial period of guanfacine exposure), first-line stimulant monotherapy (initial period of exposure), and unmedicated. Guanfacine cohort subgroups were based on previous/concurrent stimulant exposure.

***Results:*** The weight analyses included 47,910 patients (66.8% male) and the height analyses 41,248 (67.2% male). Mean initial exposure in the weight analyses was 237 days (standard deviation [SD] = 258, median = 142) for guanfacine and 257 days (SD = 284, median = 151) for first-line stimulant monotherapy, and was similar in the height analyses. Modeling indicated that guanfacine monotherapy was not associated with clinically meaningful deviations from normal *z*-score trajectories for weight (first-line, *n* = 943; nonfirst-line, *n* = 796) or height (first-line, *n* = 741; nonfirst-line, *n* = 644). In patients receiving guanfacine adjunctive to a stimulant, modeled weight (*n* = 1657) and height (*n* = 1343) *z*-scores followed declining trajectories. In this subgroup, mean standardized weight/height had decreased during previous stimulant monotherapy. For first-line stimulant monotherapy, modeled weight (*n* = 32,999) and height (*n* = 28,470) *z*-scores followed declining trajectories during year 1. In the unmedicated cohort, modeled weight (*n* = 11,515) and height (*n* = 10,050) *z*-scores were stable.

***Conclusions:*** Guanfacine monotherapy (first-line or nonfirst-line) was not associated with marked deviations from normal growth in this modeling study of children and adolescents with ADHD. In contrast, growth trajectories followed an initially declining course with stimulants, whether given alone or with adjunctive guanfacine.

## Introduction

Guanfacine extended release (GXR) is a long-acting nonstimulant treatment for patients with attention-deficit/hyperactivity disorder (ADHD) (Biederman et al. 2008b; Sallee et al. 2009b). GXR is approved for use in children and adolescents 6–17 years of age with ADHD, as a monotherapy and as adjunctive therapy to stimulants in the United States and Canada; as a monotherapy in Japan; and as a monotherapy in Europe when stimulants are not suitable, are not tolerated, or have been shown to be ineffective (Shionogi & Co. Ltd 2017; Shire Pharma Canada ULC 2019; Shire Pharmaceuticals Ltd. 2017; Shire US, Inc. 2018).

The European Medicines Agency (EMA) recommends monitoring weight and height in children and adolescents receiving stimulants for ADHD because of concerns about growth retardation (European Medicines Agency 2009, 2014). In contrast, concerns about weight gain and obesity underlie the EMA recommendation to monitor weight, height, and body mass index (BMI) regularly in patients receiving GXR (European Medicines Agency 2015; Shire Pharmaceuticals Ltd. 2017).

In two 2-year U.S. clinical trials of GXR (doses up to 4 mg/day) administered as monotherapy or adjunctive to a stimulant, mean weight, height, and BMI percentiles in children and adolescents with ADHD were stable at 12 months (Shire US, Inc. 2018), but increases in weight were reported as GXR-related treatment-emergent adverse events (TEAEs) in 7.1% (17/240) and 2.3% (6/259) of participants (Biederman et al. 2008a; Sallee et al. 2009a). In a 2-year European trial of GXR monotherapy (doses up to 7 mg/day) mean weight, height, and BMI *z*-scores remained stable throughout. At the individual level, one participant withdrew as a result of a GXR-related TEAE of weight increase, 13.0% of participants (27/207) shifted to a higher BMI category, and 8.2% (17/207) shifted to a lower category (categories were defined as <5th, ≥5th to <85th, ≥85th to <95th, and ≥95th percentile of the 2000 U.S. Centers for Disease Control and Prevention [CDC] reference population) (Huss et al. 2018).

In shorter trials, no abnormal changes in weight or height were reported at the group or individual level with GXR or guanfacine immediate release in children and adolescents with ADHD (Chappell et al. 1995; Horrigan et al. 1995; Hunt et al. 1995; Scahill et al. 2001; Biederman et al. 2008b; Sallee et al. 2009b; Hervas et al. 2014; Wilens et al. 2015; McCracken et al. 2016; Newcorn et al. 2016).

The study reported here was the first to assess the impact of pharmacotherapy with guanfacine on weight and height in children and adolescents with ADHD as observed retrospectively in a real-world clinical setting. The primary objective was to analyze longitudinal measurements of age- and gender-standardized weight and height in patients receiving any formulation of guanfacine as: first-line guanfacine monotherapy; nonfirst-line guanfacine monotherapy (following treatment with a stimulant medication); or guanfacine adjunctive to stimulant treatment. Similar analyses of patients receiving first-line stimulant monotherapy and patients not receiving ADHD pharmacotherapy provided context for the guanfacine results. *Post hoc* analyses assessed the impact of guanfacine or stimulant treatment initiation on standardized weight and height in treatment-naive patients, and the proportions of guanfacine-treated individuals with a clinically meaningful shift in standardized weight or height.

## Methods

### Data extracts and study medications

This retrospective, longitudinal, observational study used data from anonymized electronic medical records (EMRs) from the U.S. Department of Defense Military Health System (MHS), which provides health care to military families stationed in the United States and overseas. Weight and height measurements were not recorded in the MHS until October 2008, but data from January 1, 2003 to June 30, 2013 were extracted to provide historical information on diagnosis and treatment.

The primary analyses assessed the impact of guanfacine treatment regimens on change in standardized weight and height *z*-scores and were based on a data extract of EMRs that included a prescription for guanfacine (any immediate release formulation or GXR; no distinction was made between different guanfacine formulations). Subsequently, two control cohorts were derived from a separate data extract of EMRs that included an ADHD diagnosis: those with a prescription for first-line stimulant monotherapy (any formulation of amphetamine or methylphenidate) and those with no prescriptions for an ADHD medication (any formulation of guanfacine, a stimulant, or atomoxetine). ADHD was defined according to codes 314.0, 314.00, and 314.01 of the International Classification of Diseases, Ninth Revision, Clinical Modification (ICD-9-CM) 2013 (National Center for Health Statistics and the Centers for Medicare and Medicaid Services 2013).

### Study dates

The study period was January 1, 2009–June 30, 2013. The index date was defined as the first date during the study period on which patients received a prescription for a study medication (any formulation of guanfacine or a stimulant) or an ADHD diagnosis (for patients with no documented prescriptions for a study medication during the study period).

A guanfacine initiation date was also defined for patients receiving guanfacine during the study period. The baseline period was defined as the 12 months before the guanfacine initiation date for patients receiving guanfacine during the study period, or as the 12 months before the index date for all other patients. The initial period of exposure to a study medication was defined as the period between the guanfacine initiation date (for patients receiving guanfacine during the study period), or the index date (all other patients), and the date of discontinuation or change in ADHD pharmacotherapy regimen, or censoring of observations. Observations were censored when the patient reached 20 years of age, at the end of the study period, or at loss to follow-up, whichever occurred first.

In assessing discontinuation, gaps between study medication prescriptions of up to 30 days during the months of September to May and up to 121 days during the months of June to August were allowable to account for delays in renewing prescriptions and for drug holidays (structured treatment interruptions). For longer gaps, the patient was considered to have discontinued on the last day covered by the prescription that preceded the gap, under the assumption that medication was taken as indicated until all dispensed drug had been consumed.

### Study participants

Included patients had a diagnosis of ADHD (at any time), were 4–17 years of age on the index date, had an EMR extending to at least 12 months before the index date, and had no prescriptions for an ADHD medication (a study medication or any formulation of atomoxetine) in the 12 months before the index date. Included patients also had a baseline weight or height measurement (the most recent from the baseline period), and at least one eligible postbaseline measurement.

Weight and height *z*-scores adjusted for age and gender were calculated according to the 2000 CDC growth charts for children and adolescents 2–20 years of age and the methods provided by the CDC (Kuczmarski et al. 2002; Flegal et al. 2013). Eligible postbaseline measurements were within the range −4.5 ≤ *z* ≤ 4.5 (reasonableness test) and occurred during the initial period of exposure to guanfacine (for patients with a guanfacine prescription) or a stimulant (for patients with a stimulant prescription but no guanfacine prescription), or during the postbaseline period (for patients not receiving ADHD pharmacotherapy).

### Study cohorts

**FIG. 1.**
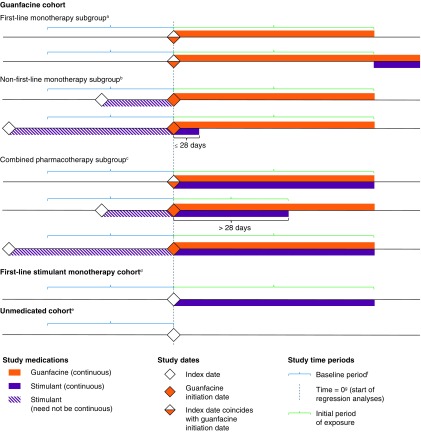
Study cohorts. ^a^First study medication is guanfacine monotherapy; subsequent stimulant prescriptions permissible. ^b^Stimulant exposure ended before or up to 28 days after first guanfacine prescription. Index date (stimulant initiation) could predate the start of the baseline period. Stimulant exposure before guanfacine initiation need not be continuous. ^c^First guanfacine prescription simultaneous with a stimulant prescription or concurrent with stimulant exposure for more than 28 days. Initial period of exposure ends at discontinuation of guanfacine and/or stimulant. Index date (stimulant initiation) could predate the start of the baseline period and stimulant exposure before guanfacine initiation need not be continuous. ^d^First study medication is stimulant monotherapy; no guanfacine prescription at any time. ^e^No prescriptions for any ADHD medication (a study medication or atomoxetine). ^f^Baseline measurement is the most recent weight or height measurement during the baseline period (the 12 months before time = 0). ^g^Guanfacine initiation date in the guanfacine cohort, index date in other cohorts. ADHD, attention-deficit/hyperactivity disorder.

Patients were assigned to one of three cohorts according to the study medications they were prescribed during the study period: guanfacine, first-line stimulant monotherapy, or unmedicated (Table 1 and Fig. 1). No matching was conducted between the cohorts. The guanfacine cohort was divided into three subgroups based on patients' stimulant exposure before or during the initial period of guanfacine exposure: first-line guanfacine monotherapy, nonfirst-line guanfacine monotherapy, and combined pharmacotherapy (Table 1 and Fig. 1).

**Table 1.** Study Cohorts and Subgroups

*Study cohort/subgroup*	*Definition*
Guanfacine cohort	At least one prescription for guanfacine (any formulation)
First-line guanfacine monotherapy subgroup	First study medication was guanfacine, with no simultaneous stimulant prescription
Nonfirst-line guanfacine monotherapy subgroup	Stimulant exposure ended before or up to 28 days after first guanfacine prescription (Fig. 1)
Combined pharmacotherapy subgroup	First guanfacine prescription was simultaneous with a stimulant prescription or concurrent with stimulant exposure for more than 28 days (Fig. 1)
First-line stimulant monotherapy cohort	At least one prescription for a stimulant but no prescription for guanfacine at any time
Unmedicated cohort	No prescribed ADHD medications

ADHD, attention-deficit/hyperactivity disorder.

### Prescribed amount and adherence

**FIG. 2.**
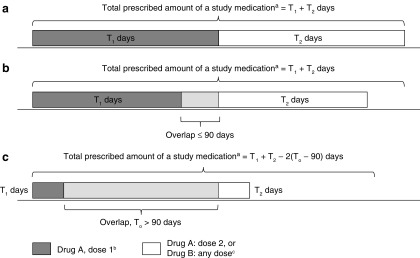
Calculation of prescribed amount (in days) of a study medication for different drugs or for different doses of the same drug: with no overlaps between prescriptions **(a)**, with overlaps ≤90 days **(b)**, or with overlaps >90 days **(c)**. ^a^Study medications: guanfacine (any formulation of GXR or GIR); stimulant (any formulations of amphetamine or methylphenidate). ^b^For example, GXR 4 mg/day. ^c^For example, GXR <> 4 mg/day or GIR any dose. GIR, guanfacine immediate release; GXR, guanfacine extended release.

The prescribed amount (in days) of a particular drug at a specified dose was defined as the sum of the length of all such prescriptions, ignoring overlaps (e.g., 4 prescriptions for GXR 4 mg/day at 30 days each = 120 days). The total prescribed amount (in days) of a study medication (i.e., guanfacine or stimulant) was defined as the sum of the prescribed amount of each particular drug at a specified dose, with overlaps of prescriptions for different drugs and/or doses capped at 90 days (Fig. 2). Adherence to a medication class was measured using the medication possession ratio (MPR), defined as the total prescribed amount (in days) of the study medication during the initial period of exposure, divided by the total number of days in the initial period of exposure, capped at 100%.

### Statistical analyses

In this retrospective database study, no *a priori* power or sample size estimates were performed and no adjustments to alpha were made to control for multiple comparisons. All *p-*values are therefore nominal.

### Analyses of longitudinal weight and height *z*-scores

Multivariable regression (random coefficients mixed-model analysis with multiple covariates [described in the following two paragraphs]) was carried out in SAS version 9.3 (SAS Institute, Inc., Cary, NC). Each cohort was analyzed separately and six models were constructed per cohort, three with weight *z*-score as the response variable and three with height *z*-score (all patients, males, and females). An unstructured covariance matrix was used for the random effects (intercept, and linear and quadratic time from the guanfacine initiation date [guanfacine cohort] or from the index date [other cohorts]). Several spatial covariance structures conceptually similar to an autoregressive structure (without the equal spacing assumption) were examined in addition to the unstructured matrix that was ultimately used. Fixed-effect predictor variables (see the following two paragraphs) represented characteristics of patients or of ADHD medications received before or during the period covered by the regression analyses. There were no issues with model convergence and the unstructured covariance model was considered adequate. No variables were removed from the models.

Analyses of the guanfacine cohort used the baseline weight or height measurement (with the time of the measurement imputed to time = 0), and all weight or height measurements recorded during the initial period of guanfacine exposure and the 60 days afterward. Each model included the following fixed-effect predictor variables: age at baseline measurement (continuous), gender (binary; “all patients” model only), guanfacine MPR during the initial period of guanfacine exposure (continuous), stimulant prescribed amount (in days) preguanfacine (continuous), use of atomoxetine before guanfacine exposure (binary), use of atomoxetine during guanfacine exposure (binary), and guanfacine cohort subgroup (categorical). The inclusion of an interaction term between time and subgroup allowed the trajectories for each subgroup to follow differently shaped paths.

Analyses of the first-line stimulant monotherapy and unmedicated cohorts used the baseline weight or height measurement (with the time of the measurement imputed to time = 0), and all weight or height measurements recorded in the initial period of stimulant exposure and the 60 days afterward (first-line stimulant monotherapy cohort), or all weight or height measurements from the index date until censoring of observations (unmedicated cohort). Each model included the following fixed-effect predictor variables: age at baseline measurement (continuous), gender (binary, “all patients” models only), and stimulant MPR during the initial period of stimulant exposure (continuous, first-line stimulant monotherapy models only).

### *Post hoc* analyses of the impact of ADHD treatment initiation on growth

The impact of ADHD treatment initiation in treatment-naive patients was assessed in separate *post hoc* regression analyses. Patients in the first-line guanfacine monotherapy subgroup, the first-line stimulant monotherapy cohort, and the unmedicated cohort were included in separate models, using the same weight or height measurements as the earlier analyses. Each model included a binary predictor variable to indicate whether a weight or height measurement was made before or on/after the index date, with linear and quadratic time at measurement as random effects.

### *Post hoc* analyses of individual weight or height *z*-score shifts in the guanfacine cohort subgroups

Individual weight or height *z*-score shifts between the baseline measurement and the last measurement included in the regression analyses were analyzed *post hoc* in the guanfacine cohort subgroups. In the absence of guanfacine-specific recommendations for potentially clinically meaningful shifts, the criteria recommended for monitoring stimulant-treated individuals in the American Academy of Child and Adolescent Psychiatry (AACAP) *Practice Parameter for the Assessment and Treatment of Children and Adolescents with ADHD* were used, namely a shift in weight or height *z*-score crossing two percentile lines on a chart showing the 5th, 10th, 25th, 50th, 75th, 90th, and 95th percentiles (Pliszka et al. 2007). Because weight and height measurements were not always concurrent, these exploratory analyses were unable to examine whether such shifts were from/to a healthy or unhealthy BMI category.

## Results

### Index date demographics and clinical characteristics

The weight analyses included 3396, 32,999, and 11,515 patients in the guanfacine, first-line stimulant monotherapy, and unmedicated cohorts, respectively, with 2728, 28,470, and 10,050 in the height analyses, respectively. Demographic characteristics at the index date were generally similar between the cohorts for the weight analyses (Table 2) and height analyses (Supplementary Table S1).

**Table 2.** Index Date Demographics and Clinical Characteristics for Patients Included in the Weight Analyses

*Patient characteristic*	*Guanfacine cohort*				*First-line stimulant monotherapy cohort*	*Unmedicated cohort*
	*All guanfacine*	*First-line guanfacine monotherapy subgroup*	*Nonfirst-line guanfacine monotherapy subgroup*	*Combined pharmacotherapy subgroup*^a^		
	N = *3396*	n = *943*	n = *796*	n = *1657*	N = *32,999*	N = *11,515*
Age, years, mean (SD)^b^	7.2 (2.9)	7.9 (3.5)	7.1 (2.7)	6.8 (2.5)	9.3 (3.5)	8.8 (3.6)
<6 years, *n* (%)^c^	1103 (32.5)	276 (29.3)	269 (33.8)	558 (33.7)	3805 (11.5)	2227 (19.3)
6–9 years, *n* (%)^c^	1702 (50.1)	410 (43.5)	393 (49.4)	899 (54.3)	16,266 (49.3)	5162 (44.8)
10–13 years, *n* (%)	423 (12.5)	169 (17.9)	106 (13.3)	148 (8.9)	7521 (22.8)	2535 (22.0)
14–17 years, *n* (%)	168 (4.9)	88 (9.3)	28 (3.5)	52 (3.1)	5407 (16.4)	1591 (13.8)
Males, *n* (%)	2497 (73.5)	666 (70.6)	591 (74.2)	1240 (74.8)	22,155 (67.1)	7353 (63.9)
Any ADHD diagnosis,^d^*n* (%)	2926 (86.2)	696 (73.8)	704 (88.4)	1526 (92.1)	31,195 (94.5)	11,515 (100.0)
Without mention of hyperactivity, *n* (%)	2378 (70.0)	441 (46.8)	599 (75.3)	1338 (80.7)	28,892 (87.6)	11,513 (100.0)
With hyperactivity, *n* (%)	2852 (84.0)	661 (70.1)	689 (86.6)	1502 (90.6)	30,476 (92.4)	11,515 (100.0)
Comorbidities that may lead to prescription of medications that affect growth present in ≥10% of patients in any cohort, *n* (%)						
Asthma, preindex date	535 (15.8)	154 (16.3)	116 (14.6)	265 (16.0)	4391 (13.3)	1630 (14.2)
Asthma, study period^e^	656 (19.3)	168 (17.8)	159 (20.0)	329 (19.9)	5057 (15.3)	1897 (16.5)
Depression, preindex date	348 (10.2)	142 (15.1)	70 (8.8)	136 (8.2)	3362 (10.2)	1269 (11.0)
Depression, study period^e^	749 (22.1)	204 (21.6)	196 (24.6)	349 (21.1)	5303 (16.1)	2137 (18.6)
Anxiety, preindex date^c^	988 (29.1)	362 (38.4)	224 (28.1)	402 (24.3)	5945 (18.0)	2027 (17.6)
Anxiety, study period^e^	1902 (56.0)	484 (51.3)	467 (58.7)	951 (57.4)	9238 (28.0)	3822 (33.2)
Autism spectrum disorder, preindex date^c^	457 (13.5)	202 (21.4)	113 (14.2)	142 (8.6)	1405 (4.3)	675 (5.9)
Autism spectrum disorder, study period^e^	862 (25.4)	281 (29.8)	213 (26.8)	368 (22.2)	2449 (7.4)	1267 (11.0)
Exposure to non-ADHD medications that may affect growth, *n* (%)						
Antiepileptics, preindex date^c^	167 (4.9)	101 (10.7)	27 (3.5)	39 (2.4)	542 (1.6)	266 (2.3)
Antiepileptics, study period^e^	407 (12.0)	153 (16.2)	109 (13.7)	145 (8.8)	1134 (3.4)	480 (4.2)
Corticosteroids, preindex date	346 (10.2)	108 (11.5)	91 (11.4)	147 (8.9)	2753 (8.3)	1049 (9.1)
Corticosteroids, study period^e^	571 (16.8)	159 (16.9)	139 (17.5)	273 (16.5)	4108 (12.4)	1657 (14.4)

^a^ Guanfacine adjunctive to a stimulant ADHD medication.

^b^ Age at index date (recorded as a continuous variable).

^c^ Differences between cohorts described in the text (not analyzed for statistical significance).

^d^ 2013 ICD-9-CM diagnosis recorded on or before index date. All other included patients had a diagnosis recorded in their EMR outside of this period.

^e^ Between index date and censoring of observations.

ADHD, attention-deficit/hyperactivity disorder; EMR, electronic medical record; ICD-9-CM, *International Classification of Diseases, Ninth Revision, Clinical Modification*; SD, standard deviation.

A lower proportion of patients receiving first-line guanfacine monotherapy had an ADHD diagnosis on or before their index date compared with those who received a stimulant on their index date, and higher proportions of patients in the overall guanfacine cohort had autism spectrum disorder or anxiety than in the other cohorts (Table 2 and Supplementary Table S1). A higher proportion of patients in the guanfacine cohort were 9 years of age or under on their index date than in the first-line stimulant monotherapy or unmedicated cohorts (82.6%, 60.8%, and 64.2%, respectively, in the weight analyses; Table 2). Similar proportions of patients were exposed to corticosteroids across the cohorts, whereas a higher proportion of patients in the guanfacine cohort than in other cohorts were exposed to antiepileptic medications (Table 2 and Supplementary Table S1).

### Exposure to ADHD medications

In the guanfacine and first-line stimulant monotherapy cohorts, the mean initial period of exposure to guanfacine or a stimulant, respectively, was ∼8 months, with high variance (standard deviation [SD] ∼9 months), giving a mean time in the analysis of ∼10 months. Patients in the guanfacine cohort with previous (or concurrent) stimulant exposure had initiated stimulant a mean of ∼12–13 months (SD ∼11 months) before initiating guanfacine (Table 3).

**Table 3.** Exposure to Study Medications for Patients Included in the Weight and Height Analyses

		*Guanfacine cohort*				*First-line stimulant monotherapy cohort*	*Unmedicated cohort*
		*All guanfacine*	*First-line monotherapy subgroup*	*Nonfirst-line monotherapy subgroup*	*Combined pharmacotherapy subgroup*		
Weight analyses	*n*	3396	943	796	1657	32,999	11,515
Time from index date to guanfacine initiation date,^a^ days before time = 0^b^	Mean (SD)	283 (338)	0 (0)	370 (343)	402 (339)	N/A	N/A
	Median (range)	148 (0–1530)	0 (—)	263 (1–1530)	326 (0–1517)		
Length of initial period of exposure,^c^ days after time = 0^b^	Mean (SD)	237 (258)	260 (278)	214 (239)	235 (254)	257 (284)	N/A
	Median (range)	142 (0–1597)	155 (0–1544)	129 (0–1385)	134 (2–1597)	151 (1–1721)	
Height analyses	*n*	2728	741	644	1343	28,470	10,050
Time from index date to guanfacine initiation date,^a^ days before time = 0^b^	Mean (SD)	285 (337)	0 (0)	369 (341)	401 (337)	N/A	N/A
	Median (range)	149 (0–1530)	0 (—)	261 (1–1530)	329 (0–1495)		
Length of initial period of exposure,^c^ days after time = 0^b^	Mean (SD)	242 (256)	265 (274)	223 (249)	238 (249)	261 (283)	N/A
	Median (range)	149 (0–1597)	167 (0–1428)	136 (0–1385)	145 (2–1597)	157 (1–1721)	

^a^ Time during which patients in the nonfirst-line guanfacine monotherapy or combined pharmacotherapy subgroup may have been exposed to stimulant medications before guanfacine initiation date.

^b^ Time = 0, guanfacine cohort: guanfacine initiation date; other cohorts: index date, that is, stimulant initiation date (first-line stimulant monotherapy cohort) or diagnosis date (unmedicated cohort).

^c^ Initial period of exposure to: guanfacine (guanfacine cohort and subgroups); stimulant (first-line stimulant monotherapy cohort).

N/A, not applicable; SD, standard deviation.

### Weight and height *z*-scores at index date and baseline

Mean weight and height *z*-scores at the index date (i.e., when ADHD-medication naive) were similar across cohorts and higher than the reference population mean (0) (not shown). By the time of the baseline measurement, weight and height *z*-scores were substantially lower in patients in the guanfacine cohort with previous (or concurrent) stimulant exposure (Table 4). For example, in the combined pharmacotherapy subgroup, mean weight *z*-score in males fell from 0.362 (95% confidence interval [0.303–0.421]) at the index date (last measurement before stimulant initiation) to −0.075 [−0.138 to −0.013] at baseline (last measurement before guanfacine initiation), and mean height *z*-score fell from 0.231 [0.165–0.298] to 0.009 [−0.057 to 0.076]. Similar trends were seen in the nonfirst-line guanfacine monotherapy subgroup (not shown).

**Table 4.** Baseline *z*-Scores and Availability of Postbaseline Measurements for Patients Included in the Weight and Height Analyses

		*Guanfacine cohort*				*First-line stimulant monotherapy cohort*	*Unmedicated cohort*
		*All guanfacine*	*First-line monotherapy subgroup*	*Nonfirst-line monotherapy subgroup*	*Combined pharmacotherapy subgroup*		
Weight analyses	*n*	3396	943	796	1657	32,999	11,515
Baseline weight *z*-score^a^	Mean (95% CI)	0.070 (0.031–0.109)	0.348 (0.270–0.426)	0.075 (−0.001 to 0.151)	–0.090 (−0.144 to −0.036)	0.419 (0.407–0.430)	0.378 (0.358–0.397)
	Percentile	53rd	64th	53rd	46th	66th	65th
Time of baseline weight measurement, days before time = 0^b^	Mean (SD)	–66.5 (72.9)	–72.9 (80.0)	–67.1 (72.1)	–62.5 (68.9)	–78.1 (82.1)	–82.8 (84.4)
Number of postbaseline^c^ measurements included in analyses							
1	*n* (%)	827 (24.4)	229 (24.3)	218 (27.4)	380 (22.9)	9078 (27.5)	2542 (22.1)
2	*n* (%)	707 (20.8)	196 (20.8)	184 (23.1)	327 (19.7)	6903 (20.9)	1823 (15.8)
3	*n* (%)	562 (16.5)	159 (16.9)	109 (13.7)	294 (17.7)	4803 (14.6)	1425 (12.4)
4 or more	*n* (%)	1300 (38.3)	359 (38.1)	285 (35.8)	656 (39.6)	12,215 (37.0)	5725 (49.7)
Height analyses	*n*	2728	741	644	1343	28,470	10,050
Baseline height *z*-score^a^	Mean [95% CI]	0.062 [0.021–0.103]	0.166 [0.084–0.247]	0.074 [−0.009 to 0.156]	–0.000 [−0.057 to 0.057]	0.198 [0.185–0.210]	0.223 [0.202–0.244]
	Percentile	52nd	57th	53rd	50th	58th	59th
Time of baseline height measurement, days before time = 0^b^	Mean (SD)	–77.9 (81.4)	–89.4 (92.3)	–77.2 (80.5)	–71.8 (75.2)	–88.6 (89.3)	–94.6 (92.3)
Number of postbaseline^c^ measurements included in analyses							
1	*n* (%)	786 (28.8)	214 (28.9)	207 (32.1)	365 (27.2)	8854 (31.1)	2678 (26.6)
2	*n* (%)	620 (22.7)	172 (23.2)	161 (25.0)	287 (21.4)	6200 (21.8)	1843 (18.3)
3	*n* (%)	447 (16.4)	137 (18.5)	79 (12.3)	231 (17.2)	4125 (14.5)	1349 (13.4)
4 or more	*n* (%)	875 (32.1)	218 (29.4)	197 (30.6)	460 (34.3)	9291 (32.6)	4180 (41.6)

^a^ Baseline *z*-score calculated from the most recent weight/height measurement made during the 12 months before guanfacine initiation date (guanfacine cohort) or index date (other cohorts).

^b^ Time = 0, guanfacine cohort: guanfacine initiation date; other cohorts: index date, that is, stimulant initiation date (first-line stimulant monotherapy cohort) or diagnosis date (unmedicated cohort).

^c^ Measurements recorded from time = 0^b^ (inclusive) until 60 days after the initial period of exposure (defined in Fig. 1), or until censoring of observations (in the unmedicated cohort).

CI, confidence interval; SD, standard deviation.

The mean time between the baseline measurement and the start of the regression analyses was ∼2–3 months across all cohorts, with high variance (SD ∼2–3 months) (Table 4).

### Availability of measurements

**FIG. 3.**
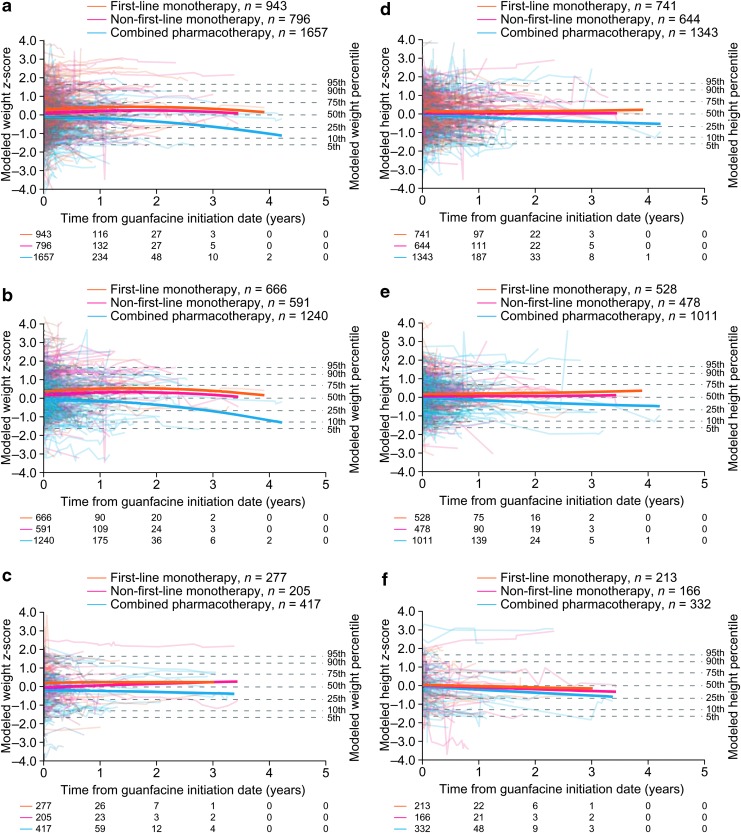
Modeled *z*-scores for weight **(a–c)** and height **(d–f)** in the guanfacine cohort for all patients **(a, d)**, males **(b, e)**, and females **(c, f)**. Solid lines show the trajectories when all other predictor variables are held constant to their overall means for patients in the guanfacine cohort, apart from “stimulant supply preguanfacine,” which is held constant to the mean of each subgroup. Traces show a random sample of patients. Numbers below the *x* axes indicate the numbers of patients with weight/height measurements on or after each time point.

**FIG. 4.**
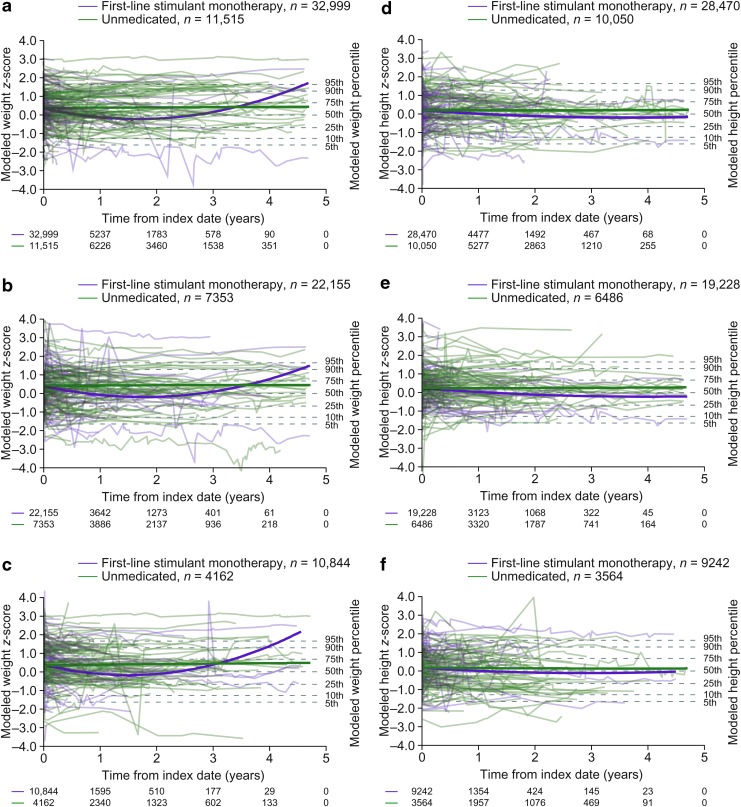
Modeled *z*-scores for weight **(a–c)** and height **(d–f)** in the first-line stimulant monotherapy and unmedicated cohorts for all patients **(a, d)**, males **(b, e)**, and females **(c, f)**. Solid lines show the trajectories when all other predictor variables are held constant to the mean of each cohort. Traces show a random sample of patients. Numbers below the *x* axes indicate the numbers of patients with weight/height measurements on or after each time point.

Approximately half of patients had at least three postbaseline measurements (Table 4), but by the end of year 1, 14.2%, 15.9%, and 54.1% of patients remained in the weight analyses in the guanfacine, first-line stimulant monotherapy, and unmedicated cohorts, respectively, with lower proportions remaining in the height analyses (Figs. 3 and 4). Trends beyond 1 year in the guanfacine and first-line stimulant monotherapy cohorts should therefore be interpreted with caution, owing to the small proportion of patients remaining in the analysis and the high rate of drop-out compared with the unmedicated cohort (see Discussion section).

### Modeled (group level) weight and height *z*-score trajectories

Guanfacine monotherapy was not associated with clinically meaningful changes in modeled weight or height *z*-score trajectories, either when administered first-line or when administered nonfirst line (Fig. 3). In the combined pharmacotherapy subgroup (guanfacine and a stimulant), modeled weight and height *z*-score trajectories followed declining courses that were significantly different in shape from those in the first-line guanfacine monotherapy subgroup, as indicated by the model interaction term (*p* < 0.0001; Tables 5, 6 and Fig. 3).

**Table 5.** Guanfacine Cohort: Mixed-Model Regression Parameter Estimates for Weight *z*-Score Models by Gender

*Model term*	*All patients,* N* = 3396*				*Males,* n* = 2497*				*Females,* n* = 899*			
	*Coefficient*	*df*	t	p	*Coefficient*	*df*	t	p	*Coefficient*	*df*	t	p
Patient characteristics												
Age at baseline measurement, years	0.01782	7189	2.70	0.0069	0.01244	5420	1.55	0.1206	0.03068	1768	2.64	0.0082
Gender^a^	−0.1453	7189	−3.41	0.0007	—	—	—	—	—	—	—	—
ADHD medication characteristics												
Guanfacine MPR during initial period of exposure to guanfacine	0.1475	7189	0.70	0.4863	0.1397	5420	0.55	0.5797	0.1684	1768	0.43	0.6702
Stimulant prescribed amount, days preguanfacine	−0.00057	7189	−7.19	<0.0001	−0.00060	5420	−6.55	<0.0001	−0.00044	1768	−2.82	0.0048
Atomoxetine use before guanfacine exposure^b^	−0.04262	7189	−0.67	0.5061	−0.03379	5420	−0.45	0.6506	−0.06186	1768	−0.49	0.6240
Atomoxetine use during guanfacine exposure^b^	−0.04696	7189	−0.82	0.4107	−0.03312	5420	−0.49	0.6210	−0.07750	1768	−0.70	0.4822
Treatment regimen												
First-line monotherapy^c^	0.2215	7189	2.92	0.0035	0.2146	5420	2.41	0.0158	0.2514	1768	1.70	0.0894
Nonfirst-line monotherapy^c^	0.1053	7189	2.11	0.0350	0.1117	5420	1.90	0.0572	0.07580	1768	0.79	0.4280
Time × treatment regimen subgroup interaction, first-line montherapy^c^	0.1812	7189	5.56	<0.0001	0.2203	5420	5.97	<0.0001	0.06687	1768	0.96	0.3354
Time × treatment regimen subgroup interaction, nonfirst-line monotherapy^c^	0.1948	7189	5.87	<0.0001	0.2166	5420	5.91	<0.0001	0.1528	1768	1.99	0.0471
Random effects												
Intercept	−0.1049	3388	−0.48	0.6332	−0.05494	2490	−0.21	0.8318	−0.3873	891	−0.92	0.3594
Time, years	−0.02123	3394	−0.77	0.4387	−0.01053	2495	−0.33	0.7379	−0.05312	897	−0.99	0.3229
Time squared	−0.05156	2568	−4.09	<0.0001	−0.06772	1888	−4.56	<0.0001	−0.00280	679	−0.14	0.8880

*p*-Values are nominal and test the null hypothesis that the coefficient of a model term = 0 (i.e., changes in the term are not related to changes in longitudinal *z*-score).

^a^ Reference = male.

^b^ Any formulation; binary variable, reference = no.

^c^ Reference = combined pharmacotherapy.

ADHD, attention-deficit/hyperactivity disorder; df, degrees of freedom; MPR, medication possession ratio.

**Table 6.** Guanfacine Cohort: Mixed-Model Regression Parameter Estimates for Height *z*-Score Models by Gender

*Model variable*	*All patients,* N* = 2728*				*Males,* n* = 2017*				*Females,* n* = 711*			
	*Coefficient*	*df*	t	p	*Coefficient*	*df*	t	p	*Coefficient*	*df*	t	p
Patient characteristics												
Age at baseline measurement, years	−0.01043	4476	−1.53	0.1255	−0.00726	3356	−0.88	0.3783	−0.01599	1121	−1.33	0.1849
Gender^a^	−0.1004	4476	−2.23	0.0260	—	—	—	—	—	—	—	—
ADHD medication characteristics												
Guanfacine MPR during initial period of guanfacine exposure	0.2083	4476	0.93	0.3513	0.02396	3356	0.09	0.9274	0.5886	1121	1.38	0.1693
Stimulant prescribed amount, days preguanfacine	−0.00041	4476	−4.96	<0.0001	−0.00044	3356	−4.64	<0.0001	−0.00031	1121	−1.90	0.0576
Atomoxetine use before guanfacine exposure^b^	−0.00543	4476	−0.08	0.9364	−0.00260	3356	−0.03	0.9733	−0.00977	1121	−0.07	0.9445
Atomoxetine use during guanfacine exposure^b^	−0.00038	4476	−0.01	0.9949	0.02061	3356	0.30	0.7635	−0.04750	1121	−0.39	0.6986
Treatment regimen												
First-line monotherapy^c^	0.01666	4476	0.21	0.8363	0.02586	3356	0.28	0.7811	−0.00496	1121	−0.03	0.9758
Nonfirst-line monotherapy^c^	0.002941	4476	0.06	0.9555	−0.00791	3356	−0.13	0.8979	0.01462	1121	0.14	0.8847
Time × therapy group interaction, first-line montherapy^c^	0.1461	4476	4.08	<0.0001	0.1574	3356	3.65	0.0003	0.09466	1121	1.63	0.1027
Time × therapy group interaction, nonfirst-line monotherapy^c^	0.1229	4476	3.42	0.0006	0.1291	3356	3.02	0.0025	0.05965	1121	1.04	0.3008
Random effects												
Intercept	0.04562	2721	0.20	0.8440	0.1960	2010	0.73	0.4680	−0.3659	703	−0.80	0.4228
Time, years	−0.1666	2726	−5.70	<0.0001	−0.1628	2014	−4.68	<0.0001	−0.1653	708	−3.40	0.0007
Time squared	0.009830	1941	0.77	0.4404	0.01186	1438	0.77	0.4422	0.001836	503	0.10	0.9204

*p*-Values are nominal and test the null hypothesis that the coefficient of a model term = 0 (i.e., changes in the term are not related to changes in longitudinal *z*-score).

^a^ Reference = male.

^b^ Any formulation; binary variable, reference = no.

^c^ Reference = combined pharmacotherapy.

ADHD, attention-deficit/hyperactivity disorder; df, degrees of freedom; MPR, medication possession ratio.

In separate analyses stratified by sex, modeled trajectories for combined pharmacotherapy remained stable in females (Fig. 3c, f) (weight, *p* = 0.3354; height, *p* = 0.1027 vs. first-line guanfacine monotherapy) and declined in males (Fig. 3b, e) (weight, *p* < 0.0001; height, *p* < 0.0001 vs. first-line guanfacine monotherapy).

Following initiation of first-line stimulant monotherapy, modeled weight *z*-scores followed a declining trajectory for the first 1–2 years (Fig. 4a–c and Table 7). Modeled height *z*-scores tended to decline for ∼3–4 years (Fig. 4d–f and Table 8). In unmedicated patients, weight and height *z*-scores remained stable over time (Fig. 4 and Tables 9, 10).

**Table 7.** First-Line Stimulant Monotherapy Cohort: Mixed-Model Regression Parameter Estimates for Weight *z*-Score Models by Gender

*Model variable*	*All patients,* N* = 32,999*				*Males,* N* = 22,155*				*Females,* N* = 10,844*			
	*Coefficient*	*df*	t	p	*Coefficient*	*df*	t	p	*Coefficient*	*df*	t	p
Patient characteristics												
Age at baseline measurement, years	0.0364	32,995	21.44	<0.0001	0.0331	22,152	15.66	<0.0001	0.0421	10,841	14.83	<0.0001
Gender^a^	−0.0454	32,995	−3.67	0.0002	—	—	—	—	—	—	—	—
ADHD medication characteristics												
Stimulant MPR during initial period of stimulant exposure	0.0058	32,995	0.35	0.7229	−0.0024	22,152	−0.12	0.9049	0.0181	10,841	0.63	0.5293
Random effects												
Intercept	0.0592	32,995	2.99	0.0028	0.0923	22,152	3.80	0.0001	−0.0470	10,841	−1.39	0.1639
Time, years	−0.7100	132,282	−108.42	<0.0001	−0.6887	89,093	−89.03	<0.0001	−0.7599	43,187	−62.02	<0.0001
Time squared	0.2127	132,282	66.09	<0.0001	0.1965	89,093	53.79	<0.0001	0.2539	43,187	38.40	<0.0001

*p-*Values test the null hypothesis that the coefficient of a model term = 0 (i.e., changes in the term are not related to changes in longitudinal *z*-score).

^a^ Reference = male.

ADHD, attention-deficit/hyperactivity disorder; df, degrees of freedom; MPR, medication possession ratio.

**Table 8.** First-Line Stimulant Monotherapy Cohort: Mixed-Model Regression Parameter Estimates for Height *z*-Score Models by Gender

*Model variable*	*All patients,* N* = 28,470*				*Males,* N* = 19,228*				*Females,* N* = 9242*			
	*Coefficient*	*df*	t	p	*Coefficient*	*df*	t	p	*Coefficient*	*df*	t	p
Patient characteristics												
Age at baseline measurement, years	−0.0023	28,466	−1.32	0.1861	−0.0004	19,225	−0.17	0.8627	−0.0060	9239	−2.05	0.0406
Gender^a^	−0.0750	28,466	−5.81	<0.0001	—	—	—	—	—	—	—	—
ADHD medication characteristics												
Stimulant MPR during initial period of stimulant exposure	0.0485	28,466	2.84	0.0046	0.0465	19,225	2.23	0.0255	0.0568	9239	1.88	0.0599
Random effects												
Intercept	0.2238	28,466	10.86	<0.0001	0.2093	19,225	8.33	<0.0001	0.1775	9239	4.99	<0.0001
Time, years	−0.2141	101,404	−33.95	<0.0001	−0.2247	68,653	−30.08	<0.0001	−0.1921	32,749	−16.39	<0.0001
Time squared	0.0296	101,404	11.50	<0.0001	0.0285	68,653	9.37	<0.0001	0.0329	32,749	6.77	<0.0001

*p-*Values test the null hypothesis that the coefficient of a model term = 0 (i.e., changes in the term are not related to changes in longitudinal *z*-score).

^a^ Reference = male.

ADHD, attention-deficit/hyperactivity disorder; df, degrees of freedom; MPR, medication possession ratio.

**Table 9.** Unmedicated Cohort: Mixed-Model Regression Parameter Estimates for Weight *z*-Score Models by Gender

*Model variable*	*All patients,* N* = 11,515*				*Males,* n* = 7353*				*Females,* n* = 4162*			
	*Coefficient*	*df*	t	p	*Coefficient*	*df*	t	p	*Coefficient*	*df*	t	p
Patient characteristics												
Age at baseline measurement, years	0.0301	11,512	11.10	<0.0001	0.0239	7351	6.77	<0.0001	0.0392	4160	9.32	<0.0001
Gender^a^	−0.0938	11,512	−4.62	<0.0001	—	—	—	—	—	—	—	—
Random effects												
Intercept	0.1410	11,512	5.27	<0.0001	0.1997	7351	5.98	<0.0001	−0.0487	4160	−1.12	0.2632
Time, years	0.0143	59,634	2.50	0.0124	0.0057	36,225	0.78	0.434	0.0293	23,377	3.17	0.0015
Time squared	−0.0002	59,634	−0.13	0.8964	0.0000	36,225	−0.02	0.9874	−0.0010	23,377	−0.30	0.7606

*p*-Values test the null hypothesis that the coefficient of a model term = 0 (i.e., changes in the term are not related to changes in longitudinal *z*-score).

^a^ Reference = male.

df, degrees of freedom.

**Table 10.** Unmedicated Cohort: Mixed-Model Regression Parameter Estimates for Height *z*-Score Models by Gender

*Model variable*	*All patients,* N* = 10,050*				*Males,* n* = 6486*				*Females,* n* = 3564*			
	*Coefficient*	*df*	t	p	*Coefficient*	*df*	t	p	*Coefficient*	*df*	t	p
Patient characteristics												
Age at baseline measurement, years	−0.0063	10,047	−2.27	0.0234	−0.0054	6484	−1.51	0.1322	−0.0076	3562	−1.72	0.0858
Gender^a^	−0.0935	10,047	−4.42	<0.0001	—	—	—	—	—	—	—	—
Random effects												
Intercept	0.3152	10,047	11.48	<0.0001	0.3031	6484	8.95	<0.0001	0.2412	3562	5.25	<0.0001
Time, years	−0.0133	42,774	−1.78	0.0751	−0.0051	26,068	−0.53	0.596	−0.0256	16,704	−2.12	0.0344
Time squared	0.0033	42,774	1.36	0.1745	0.0027	26,068	0.84	0.4004	0.0036	16,704	0.93	0.3516

*p*-Values test the null hypothesis that the coefficient of a model term = 0 (i.e., changes in the term are not related to changes in longitudinal *z*-score).

^a^ Reference = male.

df, degrees of freedom.

### Relationships after controlling for all other model variables, including time

In the guanfacine cohort, a greater prescribed amount (in days) of stimulant before initiating guanfacine was a significant predictor of lower weight *z*-scores (all patients *p* < 0.0001; males *p* < 0.0001; females *p* = 0.0048; Table 5), and also of lower height *z*-scores in all patients and in males (*p* < 0.0001; Table 6). In the first-line stimulant monotherapy cohort, stimulant adherence (as measured by MPR) was a significant predictor of higher height *z*-score in all patients and in males (*p* = 0.0046 and 0.0255, respectively; Table 8).

Guanfacine adherence, as measured by MPR, did not have a significant effect on weight or height *z*-score (e.g., all patients, weight *p* = 0.4863; height *p* = 0.3513; Table 5). Similarly, exposure to the nonstimulant atomoxetine before or during guanfacine exposure was not a significant predictor of weight or height *z*-score (Table 5).

A greater age at baseline measurement was a significant predictor of higher weight *z*-score in each of the all-patient models (guanfacine, *p* = 0.0069; first-line stimulant monotherapy, *p* < 0.0001; unmedicated, *p* < 0.0001; Tables 5, 7, and 9). Males had significantly higher weight and height *z*-scores than females (weight: guanfacine, *p* = 0.0007; first-line stimulant monotherapy, *p* = 0.0002; unmedicated, *p* < 0.0001; height: guanfacine, *p* = 0.0260; first-line stimulant monotherapy, *p* < 0.0001; unmedicated, *p* < 0.0001; Tables 5–10).

### *Post hoc* analyses of the impact of ADHD treatment initiation on growth

First-line guanfacine monotherapy was associated with significantly higher weight *z*-scores after treatment compared with before treatment in males (*p* < 0.0001) but not in females (*p* = 0.19), according to the model with the binary predictor variable (Table 11). The magnitude of the increase in weight in males was ∼0.06 of the reference population SD. First-line stimulant monotherapy was associated with significantly lower weight and height *z-*scores after treatment compared with before treatment (*p* < 0.0001, Table 11). The effect of stimulant monotherapy on weight *z*-score was greater than its effect on height *z*-score, with decreases of ∼0.14 and 0.025 of the reference population SD, respectively. In the unmedicated cohort, weight and height *z*-scores did not change significantly after ADHD diagnosis compared with before diagnosis (*p* ≥ 0.22, Table 11).

**Table 11.** Effect of Attention-Deficit/Hyperactivity Disorder Treatment Initiation on Weight and Height *z*-Scores: *Post Hoc* Regression Results

*Model*	*First-line guanfacine monotherapy subgroup*						*First-line stimulant monotherapy cohort*						*Unmedicated cohort*					
	n	*Modeled z-score at time = 0*^a^*[95% CI]; percentile*	*Coefficient*^b^	*df*	t	p	n	*Modeled z-score at time = 0*^a^*[95% CI]; percentile*	*Coefficient*^b^	*df*	t	p	n	*Modeled z-score*^a^*[95% CI]; percentile*	*Coefficient*^c^	*df*	t	p
Weight analyses																		
Males	666	0.3835 [0.2914–0.4755] 65th	0.0648	5425	3.92	<0.0001	22,155	0.4263 [0.4119–0.4408] 66th	−0.1346	171,427	−55.29	<0.0001	7353	0.4073 [0.3823–0.4323] 66th	0.004948	36,255	1.07	0.28
Females	277	0.2627 [0.1224–0.4030] 60th	0.03689	2415	1.31	0.19	10,844	0.4030 [0.3824–0.4236] 66th	−0.1417	89498	−42.22	<0.0001	4162	0.3256 [0.2930–0.3581] 63rd	0.007149	23,377	1.23	0.22
Height analyses																		
Males	528	0.1965 [0.1014–0.2916] 58th	−0.00870	3549	−0.43	0.67	19,228	0.2250 [0.2099–0.2400] 59th	−0.02418	128,010	−7.63	<0.0001	6486	0.2528 [0.2267–0.2790] 60th	0.002957	26,068	0.46	0.65
Females	213	0.08924 [−0.0645 to 0.2430] 54th	−0.06571	1516	−1.97	0.05	9242	0.1414 [0.1197–0.1631] 56th	−0.02708	65,143	−6.09	<0.0001	3564	0.1693 [0.1343–0.2043] 57th	−0.00481	16,704	−0.55	0.58

*p*-Values test the null hypothesis that the model coefficient for a binary predictor variable indicating whether measurements were made during the baseline period or on/after the index date is equal to 0.

^a^ Model intercept.

^b^ Coefficient can be interpreted as the mean change in *z*-score after treatment initiation compared with before treatment initiation, across all time.

^c^ Coefficient can be interpreted as the mean change in *z*-score after diagnosis compared with before diagnosis, across all time.

ADHD, attention-deficit/hyperactivity disorder; CI, confidence interval; df, degrees of freedom.

### *Post hoc* analyses of individual *z*-score shifts in the guanfacine cohort subgroups

A numerically higher proportion of patients receiving guanfacine monotherapy had a positive weight *z*-score shift (4.4%) than a negative shift (2.0%), in contrast to combined pharmacotherapy (positive shift 3.1%, negative shift 4.1%) (Table 12).

**Table 12.** *Post Hoc* Analyses of Individual Weight or Height *z*-Score Shifts^a^ in the Guanfacine Cohort Subgroups (*n*, %)

	*First-line monotherapy subgroup*		*Nonfirst-line monotherapy subgroup*		*Combined pharmacotherapy subgroup*	
Weight analyses	Males, *n* = 666	Females, *n* = 277	Males, *n* = 591	Females, *n* = 205	Males, *n* = 1240	Females, *n* = 417
Increase	29 (4.35%)	13 (4.69%)	26 (4.40%)	9 (4.39%)	40 (3.23%)	11 (2.64%)
Decrease	13 (1.95%)	7 (2.53%)	9 (1.52%)	6 (2.93%)	47 (3.79%)	21 (5.04%)
Height analyses	Males, *n* = 528	Females, *n* = 213	Males, *n* = 478	Females, *n* = 166	Males, *n* = 1011	Females, *n* = 332
Increase	30 (5.68%)	5 (2.35%)	16 (3.35%)	4 (2.41%)	28 (2.77%)	7 (2.11%)
Decrease	20 (3.79%)	8 (3.76%)	28 (5.86%)	11 (6.63%)	45 (4.45%)	16 (4.82%)

^a^ *z*-score shifts are defined as a change in weight or height that crosses two percentile lines on a chart showing the 5th, 10th, 25th, 50th, 75th, 90th, and 95th percentiles, based on the AACAP *Practice Parameter for the Assessment and Treatment of Children and Adolescents with ADHD* (Pliszka et al. 2007). The present analyses used the shift between the baseline measurement and the last measurement included in the regression.

AACAP, American Academy of Child and Adolescent Psychiatry; ADHD, attention-deficit/hyperactivity disorder.

## Discussion

This large database study is the first to present an analysis of longitudinal growth trajectories in children and adolescents with ADHD receiving guanfacine in a real-world clinical setting. The models covered a time period of over 3 years, but data became increasingly sparse with elongated follow-up beyond 1 year in cohorts receiving an ADHD medication and the mean analysis period was ∼10 months. Thus, trends observed during the first year of the study are more reliable than later trends.

With guanfacine monotherapy, administered first-line or following stimulant treatment, modeled standardized weight and height *z*-score trajectories followed those of the reference population norms (i.e., were approximately horizontal) (Kuczmarski et al. 2002). A *post hoc* model indicated that treatment-naive males had significantly higher weight z-scores after starting guanfacine, compared with before. This group-level increase equates to a gain of 0.8 kg for a 17-year-old male and less for younger children (Kuczmarski et al. 2002), and would not qualify as a *z*-score shift based on recommendations from AACAP (see Methods section) (Pliszka et al. 2007).

A separate *post hoc* analysis of individual weight *z*-score shifts based on the AACAP recommendations showed a potentially clinically meaningful increase in 4.4% of patients receiving guanfacine monotherapy, and a potentially clinically meaningful decrease in 2.0%. The proportion with an increase was of a similar order of magnitude to the proportion of patients in long-term clinical trials for whom GXR-related TEAEs of weight increase were reported (Biederman et al. 2008a; Sallee et al. 2009a; Huss et al. 2018).

In the first-line stimulant monotherapy cohort, modeled standardized weight trajectory declined during the first 1–2 years of treatment, with a less pronounced decline in standardized height, consistent with the known side effects of stimulant medications. A *post hoc* model indicated that individuals in the first-line stimulant monotherapy cohort had significantly lower weight and height *z*-scores after initiating stimulant therapy, compared with before. These group-level deficits were equivalent to ∼1.6 kg and 0.2 cm at the age of 17 years (Kuczmarski et al. 2002) and would not qualify as a potentially clinically meaningful *z*-score shift based on the AACAP recommendations (Pliszka et al. 2007).

The initial trends in the first-line stimulant monotherapy cohort are consistent with the results of a quantitative analysis of 20 longitudinal studies of children and adolescents with ADHD receiving stimulant monotherapy with at least 1 year of follow-up, in which the authors concluded that stimulant treatment was associated with slower-than-expected increases in weight and height, an effect that attenuated over time (Faraone et al. 2008). Similarly, more individuals shifted to a lower than to a higher weight, height, or BMI *z*-score category in a recently published 2-year trial of open-label lisdexamfetamine dimesylate (Coghill et al. 2017; Banaschewski et al. 2018). Long-term observational follow-up of participants in the Multimodal Treatment Study of children with ADHD (*n* = 515) and controls (*n* = 289) indicates that extended use of stimulants is associated with suppression of adult height at 25 years of age (Swanson et al. 2017). In the present study, the fact that greater stimulant adherence (as measured by MPR) was a significant predictor of higher height *z*-scores in the first-line stimulant monotherapy cohort may indicate active management by clinicians (i.e., fewer structured treatment interruptions occurred if potential height deficits were not a concern).

In a patient population with reduced mean weight and height *z*-scores following previous stimulant monotherapy, modeled standardized weight and height followed declining trajectories after augmentation of stimulant treatment with guanfacine. The continued decline of the model trajectories may, in part, be due to the imputation of the baseline measurement to time = 0. For individuals whose *z*-score decreased between their baseline measurement and augmentation of the stimulant with guanfacine, the imputation would lead to a steeper postbaseline decline than if the time of the baseline measurement had been included in the model.

In patients with ADHD who did not receive pharmacotherapy, modeled standardized weight and height trajectories remained stable over time, suggesting that ADHD itself was not associated with dysregulated growth at the group level. In a study from the 1990s, a small attenuation in height *z*-score was reported in 124 children and adolescents with ADHD compared with healthy controls; this resolved by late adolescence and was reported to be unrelated to exposure to ADHD medications (Spencer et al. 1996, 1998), although others have commented that the study is difficult to interpret owing to lack of information on the length of time on treatment (Poulton 2005). The results of the present study are consistent with those of a prospective longitudinal study of children and adolescents with ADHD (*n* = 137) and matched controls (*n* = 124) who were followed up to a mean age of 22 years. In that study, the investigators concluded that any delays in expected growth had resolved by adulthood (Biederman et al. 2010).

Standardized mean weight in treatment-naive children and adolescents with ADHD in the present study was higher than the population norm, and the models indicated that older patients tended to have higher standardized weight than younger patients. These findings could reflect both the high prevalence of obesity in children and adolescents with ADHD (Waring et al. 2008) and the fact that the most recent data used to generate the reference population norm *z*-scores are from 1980 (Kuczmarski et al. 2002), so do not capture the increasing prevalence of obesity overall and with age in the general U.S. and MHS database populations (Eilerman et al. 2014; National Center for Health Statistics 2016; Skinner et al. 2018).

Strengths of this retrospective review include the availability of large numbers of real-world patient records spanning long time periods. The first-line stimulant monotherapy and unmedicated cohorts were much larger than in previous studies of longitudinal weight or height *z*-scores in children and adolescents with ADHD (Faraone et al. 2008; Dura-Trave et al. 2012; Kim et al. 2014). In addition, the majority of patients were 9 years of age and under at the index date (Table 2 and Supplementary Table S1), allowing weight and height to be assessed during a period of rapid growth. Moreover, the study population contained a broader sample of patients than were included in long-term clinical trials of GXR monotherapy (Biederman et al. 2008a; Sallee et al. 2009a; Huss et al. 2018), for example, patients 4–5 years of age, those with psychiatric comorbidities, and those of all weights. Finally, universal access to care in the MHS may favor compliance with medications.

A number of caveats, however, should be noted regarding the patient population. First, care should be taken in applying these results in other jurisdictions, given that the ICD diagnostic criteria used in the present study identify a narrower subset of severely affected individuals than the *Diagnostic and Statistical Manual of Mental Disorders* (DSM) *Fourth Edition* (American Psychiatric Association 2000) or *DSM Fifth Edition* (American Psychiatric Association 2013) criteria (Santosh et al. 2005; National Institute for Health and Care Excellence 2018) and compliance with prescribed medications may be different in non-MHS patients. Second, children with potential growth/maturation issues may have been more likely to have baseline and longitudinal weight or height measurements and more frequent health care visits, and therefore to be included in the analyses, than children without. The same is true of younger children, compared with older children/adolescents.

Third, children of military personnel tend to be younger and to have a lower prevalence of obesity than children in the general U.S. population (United States Census Bureau 2016; Eilerman et al. 2014; U.S. Department of Defense 2014). Finally, deployment of a military parent for extended periods of time is known to be associated with reduced frequency of switching between ADHD medications and increased frequency of mental/behavioral health care visits compared with when the parent is at home (Hisle-Gorman et al. 2014).

The study design and type of models employed also lead to limitations in interpretation. First, the models did not control for some factors that may have affected outcomes, in particular medications known to affect growth (Table 2 and Supplementary Table S1). Inhaled corticosteroid use in children with mild-to-moderate asthma leads to reduced height gain, with a trend toward greater reductions at higher doses (Loke et al. 2015), and height deficits persist into adulthood (Childhood Asthma Management Program Research Group et al. 2000; Kelly et al. 2012). With the antiepileptic medications most commonly prescribed in the guanfacine cohort, valproate/valproic acid and oxcarbazepine, weight gain is a commonly reported concern (Verrotti et al. 2009; Petty et al. 2014; Hamed 2015; Garoufi et al. 2016), and valproate has also been reported to reduce height gain (Lee et al. 2013). The design of present analyses, however, meant that time-varying covariates, such as exposure to other medications, could not be included. Moreover, medication dosage and route of administration were not captured and asthma severity is not coded in the ICD-9-CM system (National Center for Health Statistics and the Centers for Medicare and Medicaid Services 2013). Other factors not controlled for included comorbidities, ADHD severity, ADHD medication dosage, parental military rank, periods of parental deployment, ethnicity, and location. In particular, compared with the other cohorts, the guanfacine cohort contained higher proportions of individuals with conditions for which drugs associated with antipsychotic weight gain may be prescribed (Musil et al. 2015), such as depression, anxiety, and autism spectrum disorders (Table 2 and Supplementary Table S1).

Second, calculated *z*-scores for individuals whose weight or height lie outside the CDC reference population 3rd and 97th percentiles (*z* = ± 1.88) are known to be unreliable (Kuczmarski et al. 2002; Flegal et al. 2009, 2013).

Third, the models could not distinguish between stimulant use before the baseline measurement and stimulant use between the baseline measurement and guanfacine initiation date because the measurement was imputed to time = 0.

Fourth, the sample included patients whose data could not contribute to the trajectory analyses because their only postbaseline measurement also occurred at time = 0. This may have affected up to a quarter of patients receiving pharmacotherapy (Table 4), if their weight and height were measured on the day they were first prescribed a new study medication.

Fifth, the first-line stimulant monotherapy cohort did not include patients who later received guanfacine, so the trends observed may not apply to all treatment-naive children and adolescents with ADHD who start stimulant treatment. In particular, patients for whom first-line stimulant monotherapy was ineffective, poorly tolerated, or impacted on growth or cardiovascular health are more likely to appear in the combined pharmacotherapy or nonfirst-line guanfacine monotherapy subgroups than in the first-line stimulant monotherapy cohort.

Finally, the cohorts were not included in the same model. This means that they cannot be compared directly—for example, between-cohort differences, such as the younger mean age in the guanfacine cohort, could not be controlled for.

Another limitation is the high level of drop-out, meaning that trends at later time points were based on far fewer patients than trends at earlier time points. Although longitudinal regression results are minimally affected by randomly occurring drop-out, they can become seriously biased when drop-out is predicted by baseline or response variables (Gustavson et al. 2012). In the present study, the drop-out rate was higher in the medicated cohorts than in the unmedicated cohorts (Figs. 3 and 4), suggesting that medication use affected the likelihood of drop-out, and therefore indicating potential for bias. Furthermore, the risk of bias is especially great when the response variable (in this case weight or height *z*-score) for those remaining is within an “acceptable” range as time progresses (Howell 2007). In the present study, the possibility of treating physicians discontinuing or switching patients' medication because of growth concerns represents a second source of potentially serious bias in the regression models.

## Conclusions

Guanfacine monotherapy given first-line or following a stimulant was not associated with marked deviation from a normal growth trajectory at the group level in this large, retrospective regression modeling study of children and adolescents with ADHD. At the individual level, fewer than 5% of patients had a potentially clinically meaningful increase in weight, based on z-score shifts. The study confirmed that stimulant therapy, with or without guanfacine augmentation, is associated with slower-than-expected growth during the first year of treatment at the group level.

## Clinical Significance

These findings support current recommendations for regular monitoring of patients' weight and height to assess the potential impact of ADHD treatment regimens on growth.

## Supplementary Material

Supplemental data
