# Supplementary material for: Weight and Height in Children and Adolescents with Attention-Deficit/Hyperactivity Disorder: A Longitudinal Database Study Assessing the Impact of Guanfacine, Stimulants, and No Pharmacotherapy
Source: J Child Adolesc Psychopharmacol. 2019 May 13;29(4):285–304. doi: 10.1089/cap.2018.0132 (PMC6534094; doi:10.1089/cap.2018.0132)
Supplement: Supplemental data [file Supp_Table1.pdf]

Supplementary Data

SUPPLEMENTARY TABLE S1. INDEX DATE DEMOGRAPHICS AND CLINICAL CHARACTERISTICS FOR PATIENTS INCLUDED IN THE HEIGHT ANALYSES

	Guanfacine cohort					
Patient characteristic	*All guanfacine* N = *2728*	*First-line guanfacine monotherapy subgroup* n = *741*	*Nonfirst-line guanfacine monotherapy subgroup* n = *644*	*Combined pharmacotherapy subgroup*[a] n = *1343*	*First-line stimulant monotherapy cohort* N = *28,470*	*Unmedicated cohort* N = *10,050*
Age, years, mean (SD)[b]	7.2 (2.9)	8.0 (3.5)	7.0 (2.7)	6.8 (2.5)	9.3 (3.5)	8.9 (3.6)
<6 years, *n* (%)[c]	906 (33.2)	217 (29.3)	224 (34.8)	465 (34.6)	3324 (11.7)	1955 (19.5)
6–9 years, *n* (%)[c]	1343 (49.2)	321 (43.3)	308 (47.8)	714 (53.2)	13,894 (48.8)	4441 (44.2)
10–13 years, *n* (%)	331 (12.1)	128 (17.3)	88 (13.7)	115 (8.6)	6439 (22.6)	2201 (21.9)
14–17 years, *n* (%)	148 (5.4)	75 (10.1)	24 (3.7)	49 (3.6)	4813 (16.9)	1453 (14.5)
Males, *n* (%)	2017 (73.9)	528 (71.3)	478 (74.2)	1011 (75.3)	19,228 (67.5)	6486 (64.5)
Any ADHD diagnosis,[d] *n* (%)	2349 (86.1)	547 (73.8)	568 (88.2)	1234 (91.9)	26,965 (94.7)	10,050 (100.0)
Without mention of hyperactivity, *n* (%)	1910 (70.0)	343 (46.3)	484 (75.2)	1083 (80.6)	25,031 (87.9)	10,048 (100.0)
With hyperactivity, *n* (%)	2292 (84.0)	521 (70.3)	556 (86.3)	1215 (90.5)	26,355 (92.6)	10,050 (100.0)
Comorbidities that may lead to prescription of medications that affect growth present in ≥10% of patients in any cohort, *n* (%)						
Asthma, preindex date	437 (16.0)	127 (17.1)	101 (15.7)	209 (15.6)	3876 (13.6)	1453 (14.5)
Asthma, study period[e]	518 (19.0)	134 (18.1)	123 (19.1)	261 (19.4)	4371 (15.4)	1692 (16.8)
Depression, preindex date	280 (10.3)	112 (15.1)	55 (8.5)	113 (8.4)	2898 (10.2)	1107 (11.0)
Depression, study period[e]	595 (21.8)	152 (20.5)	156 (24.2)	287 (21.4)	4524 (15.9)	1880 (18.7)
Anxiety, preindex date[c]	794 (29.1)	288 (38.9)	174 (27.0)	332 (24.7)	5105 (17.9)	1785 (17.8)
Anxiety, study period[e]	1513 (55.5)	383 (51.7)	365 (56.7)	765 (57.0)	7925 (27.8)	3336 (33.2)
Autism spectrum disorder, preindex date[c]	366 (13.4)	157 (21.2)	95 (14.8)	114 (8.5)	1227 (4.3)	592 (5.9)
Autism spectrum disorder, study period[e]	704 (25.8)	228 (30.8)	173 (26.9)	303 (22.6)	2120 (7.4)	1119 (11.1)
Exposure to non-ADHD medications that may affect growth, *n* (%)						
Antiepileptics, preindex date[c]	130 (4.8)	79 (10.7)	20 (3.1)	31 (2.3)	461 (1.6)	235 (2.3)
Antiepileptics, study period[e]	318 (11.7)	117 (15.8)	82 (12.7)	119 (8.9)	960 (3.4)	428 (4.3)
Corticosteroids, preindex date	264 (9.7)	87 (11.7)	72 (11.2)	105 (7.8)	2397 (8.4)	918 (9.1)
Corticosteroids, study period[e]	427 (15.7)	118 (15.9)	99 (15.4)	210 (15.6)	3492 (12.3)	1454 (14.5)

[a]Guanfacine adjunctive to a stimulant ADHD medication.
[b]Age at index date (recorded as a continuous variable).
[c]Differences between cohorts described in the text (not analyzed for statistical significance).
[d]2013 ICD-9-CM diagnosis recorded on or before index date. All other included patients had a diagnosis recorded in their EMR outside of this period.
[e]Between index date and censoring of observations.
ADHD, attention-deficit/hyperactivity disorder; EMR, electronic medical record; ICD-9-CM, *International Classification of Diseases, Ninth Revision, Clinical Modification*; SD, standard deviation.
